# Study on the Electrorheological Ultra-Precision Polishing Process with an Annular Integrated Electrode

**DOI:** 10.3390/mi12101235

**Published:** 2021-10-12

**Authors:** Cheng Fan, Yigang Chen, Yucheng Xue, Lei Zhang

**Affiliations:** Jiangsu Provincial Key Laboratory of Advanced Robotics, Soochow University, Suzhou 215021, China; chfan@suda.edu.cn (C.F.); ygchenygchen@stu.suda.edu.cn (Y.C.); 20185229045@stu.suda.edu.cn (Y.X.)

**Keywords:** electrorheological polishing, polishing tool, roughness, integrated electrode

## Abstract

Electrorheological (ER) polishing, as a new ultra-precision super-effect polishing method, provides little damage to the workpiece surface and is suitable for polishing all kinds of small and complex curved surface workpieces. In this paper, an ER polishing tool with an annular integrated electrode is developed. The orthogonal experiments are carried out on the six influencing factors of ER polishing which include the applied voltage, the abrasive particle size, the abrasive concentration, the polishing gap, the polishing time and the tool spindle speed. The influence order of these six factors on the ER polishing is obtained. On this basis, the effect of a single process parameter of ER polishing on surface roughness is studied experimentally.

## 1. Introduction

With the development of communications technology and the modern medical level, there is an increasing demand for electronic and optical components with complex surfaces and high surface quality, such as the spherical and aspheric lenses [1]. In the traditional process, most aspheric lenses are made by cemented carbide mold. These molds not only need higher geometric accuracy but also require lower surface roughness. Therefore, after the mold is made, the polishing process must be carried out to ensure the surface quality of the mold [2]. As the final step in the manufacturing process, polishing is used for the machining of plane, mirror and free-form surface, which ultimately affect the quality of the workpiece [3,4,5]. In the traditional polishing methods, the polishing pad and grinding head are mostly used to directly contact with the workpiece surface for polishing, and grinding paste is added between the polishing pad and the workpiece, or abrasive particles such as abrasive belt and grinding wheel are fixed on the grinding head [6,7]. When the polishing head is used to polish the complex and small mold, the traditional processing technology cannot make the polishing head suitable for the small mold due to the limitation of the tool size. On the other hand, the polishing head with fixed abrasive wears easily [8]. With the reduction of the number of effective cutting edges and the decrease of the shear speed, the surface roughness of the workpiece becomes uneven [9,10]. In order to overcome the defects of traditional polishing, it is necessary to seek a flexible and compliant polishing process that can continuously gather the abrasive particles in the polishing area for a long time. People have begun to study and use non-traditional polishing methods to achieve a constant material removal rate, including laser polishing [11,12], ultrasonic polishing [13,14,15], electrochemical polishing [16,17,18], solid jet polishing [19,20,21,22,23], liquid suspension polishing [24,25], etc.

ER polishing is a new type of compliant ultra-precision polishing methods. The ER fluid is a kind of suspension composed of solid particles (dispersed phase) with high dielectric constant and liquid (continuous phase) with good insulation performance. The ER effect occurs under the action of an electric field. The apparent viscosity increases with the increase of electric field strength, showing obvious shear yield strength. When the electric field intensity decreases to zero, the viscosity of ER fluid decreases and returns to the initial value. The ER polishing technology is a compliant polishing method which uses the ER effect under an electric field to process the workpiece surface. It is suitable for both conductive and non-conductive workpieces [26]. The controllable flexible polishing head is generated by the ER effect to realize the adaptive polishing of complex surfaces. The complex surface can be polished by controlling the voltage, tool electrode speed and polishing path, improving the processing quality.

Since the emergence of the ER polishing technology, many researchers have been trying to research and explore it. At present, research on ER polishing mainly focuses on the process method, process law, tool system and corresponding equipment development of polishing micro-aspheric lenses of optical glass and their forming die made of cemented carbide, silicon wafer and other conductive and non-conductive workpieces [27,28,29]. Zhang et al. [30,31,32] studied the mechanism and material removal model of ER polishing, regarding tool electrode speed, processing time, applied voltage, abrasive type and concentration. Hui et al. [33] applied torque sensor and speed sensor to study the shear characteristics of ER polishing and greatly reduced the surface roughness of K9 glass by polishing. Luo et al. [34] polished the surface of optical glass with the ER fluid containing ferric oxide abrasive and explained the influence of polishing pressure and slurry shear rate on the final material removal rate. However, due to the poor practicability of ER polishing tool design and low material removal rate, ER polishing is still not widely used in industry. In this paper, a new tool system of ER polishing is designed and developed, which contains an annular integrated electrode. The tool system integrates the cathode and anode that produce the ER effect but insulates them from each other. The proposed ER polishing tool system can be installed on the machine center as a module, and the machining materials are not limited by conductors or non-conductors.

The remainder of the paper is organized as follows. Section 2 introduces a new developed ER tool system. Section 3 shows the experiments and discussion. Conclusions are presented in Section 4.

## 2. ER Polishing Equipment

**Figure 1:**
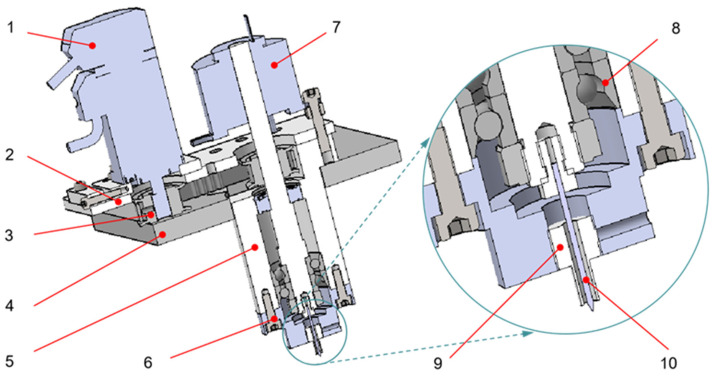
Structure diagram of integrated ER polishing tool system. (1) Driving motor, (2) Moving slide plate, (3) Synchronous pulley, (4) Support plate, (5) Outer sleeve, (6) Connection flange, (7) Conductive slip ring, (8) Deep groove ball bearing, (9) Annular electrode, (10) Tool needle.

**Figure 2:**
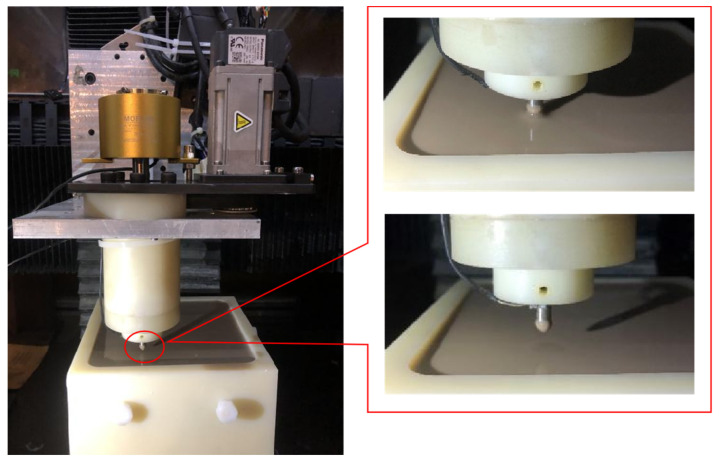
ER polishing equipment and flexible polishing head due to ER effect.

Figure 1 shows the structure of the self-made integrated ER polishing tool system. The tool system is composed of the motor, the support plate, the sliding plate, the synchronous pulley, the synchronous belt, the conductive slip ring, the outer sleeve, the deep groove ball bearing, the angular contact bearing, the tool shaft, the tool needle, the lock nut, the connecting flange and the annular electrode. Figure 2 shows the ER polishing equipment and the flexible polishing head due to ER effect.

The motor drives the tool shaft to rotate through the synchronous wheel and synchronous belt. The tool shaft is equipped with conductive slip ring. The outer ring of conductive slip ring is fixed with the support plate by bolts and nuts. The inner ring and tool shaft cooperate to transmit electricity to the tool shaft and the tool pin and rotate with the tool shaft synchronously. The tool pin is connected to the tool shaft by a thread and rotates with the tool shaft. The annular electrode is installed on the connecting flange in a static state. During ER polishing, the positive pole of the power is supplied to the annular electrode, and the negative power is supplied to the terminal of the conductive slip ring. By immersing the tool end into the ER polishing liquid mixed with polishing abrasive particles, the high voltage electric field is formed between the tool needle and the annular electrode, which causes the ER fluid mixed with polishing abrasive particles near the tool end to produce the ER effect. As a result, a soft flexible polishing head is formed at the tip of needle electrode (as shown in Figure 2). The rotation of the needle electrode drives the movement of the polishing abrasive particles to realize the micro removal of the material on the workpiece surface.

Different from the traditional ER polishing tool which needs auxiliary electrodes to construct electric field, the polishing tool with an annular electrode can construct an electric field by itself. When the polishing tool is working, the annular electrode is connected to the positive pole, and the tool needle is connected to the negative pole. As a result, an electric field is formed between the annular electrode and the tool needle. When people polish different materials, they do not need to redesign the polishing tool or add auxiliary electrodes.

## 3. Experiments and Discussion

In this paper, the key factors affecting ER polishing are explored by orthogonal experimental method, and then, the influence of a single factor on polishing is studied. The ER fluid used in the experiments is composed of starch particles as disperse phase, silicone oil as continuous phase and diamonds as abrasive particles. When the composition of ER fluid and abrasive particles are determined, the main factors affecting polishing are applied voltage, abrasive particle size, abrasive concentration, polishing gap, polishing time and tool spindle speed.

**Figure 3:**
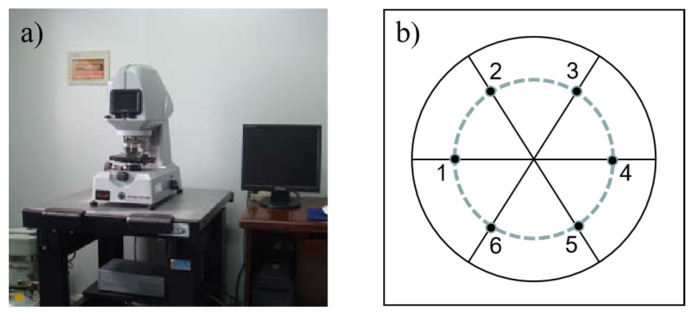
(**a**) The measuring equipment and (**b**) the distribution of the measuring points.

In the experiments, aluminum is used as the workpiece. Before ER polishing, hand-held pneumatic polishing machines were used to pre-polish the surface of the workpiece, making the surface roughness reach 0.2μm. In both orthogonal experiments and single factor experiments, the polished area is a circle with a radius of 5 mm, and the recorded data is the average roughness of the six measuring points. The measurement of the roughness is carried out on the WYKO NT1100, as shown in Figure 3a. The distribution of the six-measuring points is shown in Figure 3b.

### 3.1. Orthogonal Experiments of ER Polishing

In the orthogonal experiment, each factor is divided into three levels. The applied voltages are 1000, 2000 and 3000 V. The abrasive particles used for polishing are diamond abrasive particles, and the polishing grinding particle sizes are W1, W5 and W10, respectively. The polishing abrasive particle concentrations are fixed as 5%, 10% and 15%, respectively. The processing gaps are 0.3, 0.5 and 1 mm respectively. The polishing times are set as 10, 20 and 30 min, respectively. The speeds of the polishing spindle are 1000, 2000 and 3000 r/min, respectively. The surface roughness after polishing is the index which is used to describe the polishing effect. In order to verify the influence of the various factors on the surface roughness after polishing, an orthogonal table *L_18_* (3^6^) with 6 factors and 3 levels is designed for orthogonal experiment. The distribution of the factors and the levels for the ER polishing experiments are shown in Table 1.

The aforementioned equipment and polishing tools are used to carry out 18 relevant experiments. The experimental scheme, experimental results and SPSS analysis and processing results are shown in Table 2.

Firstly, the sum and average of the roughness corresponding to the same level of each factor should be calculated. For example, in Table 2, when the applied voltage is 1000 V which is the first level in Table 1, the corresponding roughness values are 0.1702, 0.1268, 0.1156, 0.0908, 0.1612 and 0.1042 μm, respectively, and the average value *k_1_* is 0.128 μm. When the input voltage is 2000 V, the average roughness *k_2_* is 0.097; when the input voltage is 3000 V, the average surface roughness *k_3_* is 0.075. For the factor of input voltage, the range value, which is the difference between the maximum and minimum values of *k*_1_, *k*_2_ and *k*_3_, is 0.128 − 0.075 = 0.053. The ranges of other factors in Table 2 can also be calculated according to this method. The range indicates the influence degree of the factor on the final index. The greater the range of the factor, the greater the influence of the factor on the index. After analyzing the range values of the orthogonal experimental results, it can be seen from Table 2 that the order of influence factors on the surface roughness after ER polishing is as follows: applied voltage, tool spindle speed, polishing time, polishing gap, abrasive particle size and abrasive concentration. The purpose of optimizing parameters in this paper is to reduce the roughness value. Therefore, the smaller the value of *k*, the better the polishing effect. Therefore, we choose the parameter value according to *k*. Then the best combination scheme is selected as A3F3E3D1B3C2. In the optimized scheme selected, the applied voltage is set to 3000 V, the tool spindle speed is set to 3000 r/min, the polishing time lasts for 30 min, the polishing gap is 0.3 mm, the abrasive particle size is selected as W10, and the abrasive concentration is 10%.

**Figure 4:**
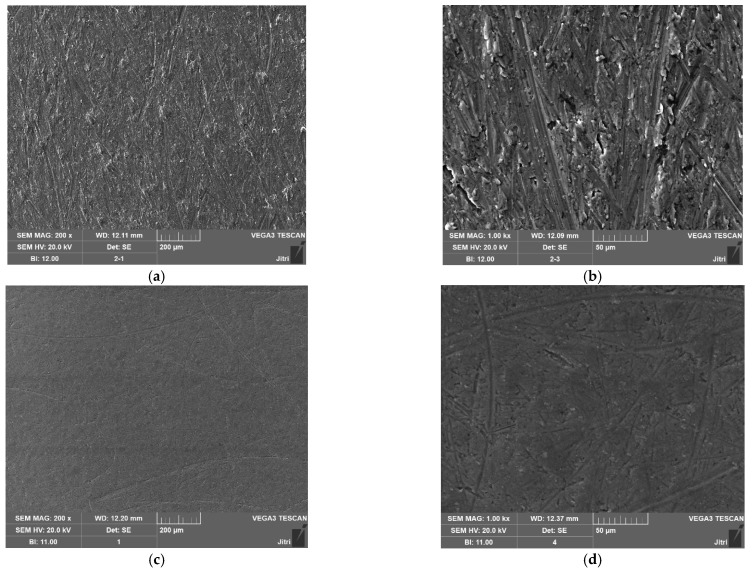
Surface morphology before and after ER polishing. (**a**) The enlarged surface before polishing (Scale: 200 μm). (**b**) The enlarged surface before polishing (Scale: 50 μm). (**c**) The enlarged surface after polishing (Scale: 200 μm). (**d**) The enlarged surface after polishing (Scale: 50 μm).

The ER polishing experiments are carried out in the best experimental combination, and the concentration of dispersed phase remains unchanged. The experimental results are shown in Figure 4. Figure 4a,b is an image enlarged by using tungsten filament scanning electron microscopy after pre-rough polishing, while Figure 4c,d is an image of surface after ER polishing. In Figure 4a,b, it can be found that there are some bumps on the workpiece surface, which are well improved in Figure 4c,d, indicating that the ER polishing has a good effect on removing the scratches and bumps on the workpiece surface. Although some small scratches can be observed on the polished surface, large scratches have been removed. Small scratches are caused by the abrasive particles that constantly rotate and hit the surface of the workpiece.

### 3.2. Single Factor Experiments of ER Polishing

In order to further explore the effects of the applied voltage, abrasive particle size, polishing abrasive concentration, polishing gap, polishing time and spindle speed on the polished roughness of the workpiece surface, single factor experiments of ER polishing were carried out. The material of the workpiece was aluminum, and the surface roughness was 0.2μm before polishing. In the experiments, we changed the value of a single factor, and the comparative experiments were carried out with three different polishing gaps for each factor to explore the influence law of a single factor.

Firstly, the initial applied voltage was set to 1000 V, the size of the abrasive particle was W1 and the concentration of the polishing slurry was 5%. Additionally, the polishing gap between the tool and surface was kept as 1 mm, the spindle speed was set to 1000 r/min and the polishing lasted for 20 min.

**Figure 5:**
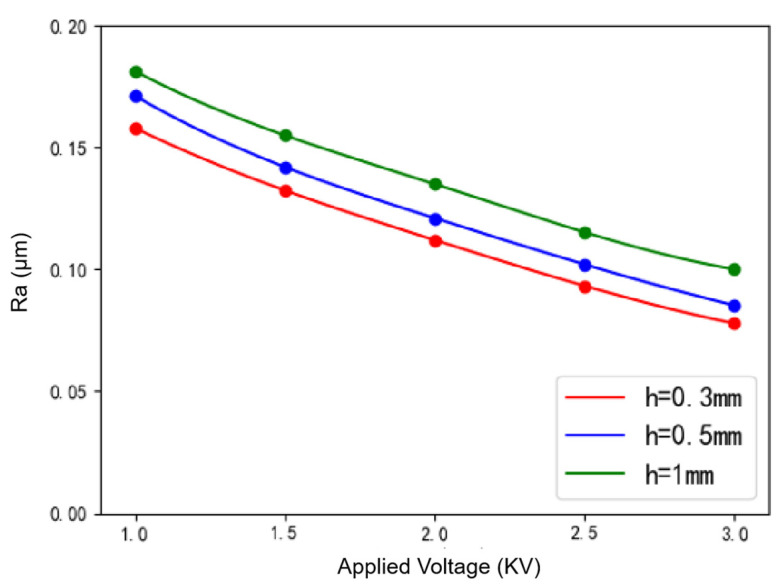
Variation of surface roughness with voltage under various polishing gaps.

The variation of workpiece surface roughness with voltage under various polishing gaps is shown in Figure 5. As indicated by Figure 5, when the voltage increases two times from 1000 to 3000 V, the roughness decreases by 52.5% when *h* = 0.3 mm and 48.1% when *h* = 0.5 mm. When the applied voltage is low, the ER effect is weak, the “hardness” of the flexible polishing head is relatively low, and the polishing pressure on the workpiece is also not high enough. As a result, the phenomenon of material removal is not obvious. With the increase of the applied voltage, the ER effect is enhanced, and the bonding force between particles and the slurry viscosity increases. What is more, the abrasive aggregation effect is more obvious. That is to say, with the increase of the number of abrasives involved in polishing, the material removal ability of the polishing head is gradually enhanced, and the polishing effect is better.

**Figure 6:**
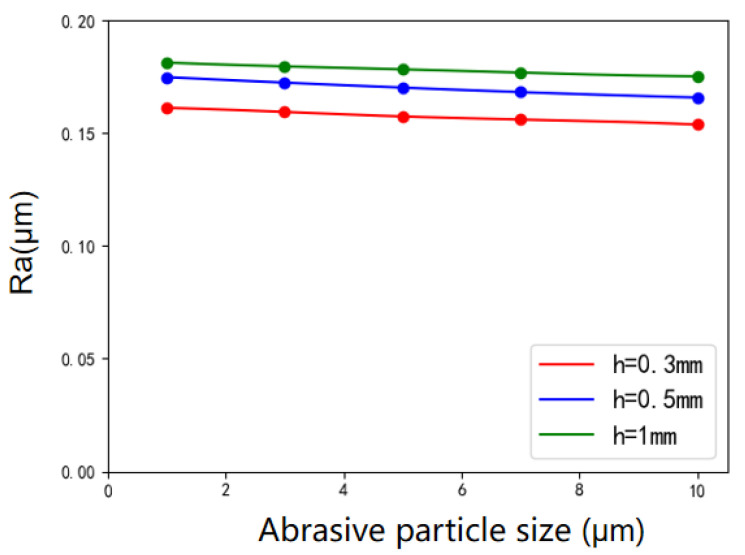
Variation of surface roughness with abrasive particle size under various polishing gaps.

The variation trend of surface roughness with abrasive particle size is shown in Figure 6. It can be seen that when the size of the diamond abrasive gets larger, the surface roughness value decreases. When the size of the diamond abrasive increases from 1 to 10 µm, the roughness value decreases by 7.88% when *h* = 0.3 mm and 5.71% when *h* = 0.5 mm. The abrasive particles adhere to the chain of particles composed of dispersed particles, and the radius of the abrasive particles is much smaller than that of dispersed particles. Because the polishing tool head used in the experiment is small and the number of abrasive particles gathered at the end of the polishing tool head is limited, the actual effect of the abrasive particle size on the polishing is not obvious; thus, the variation of the surface roughness of the workpiece after polishing with different abrasive particle sizes does not change significantly.

**Figure 7:**
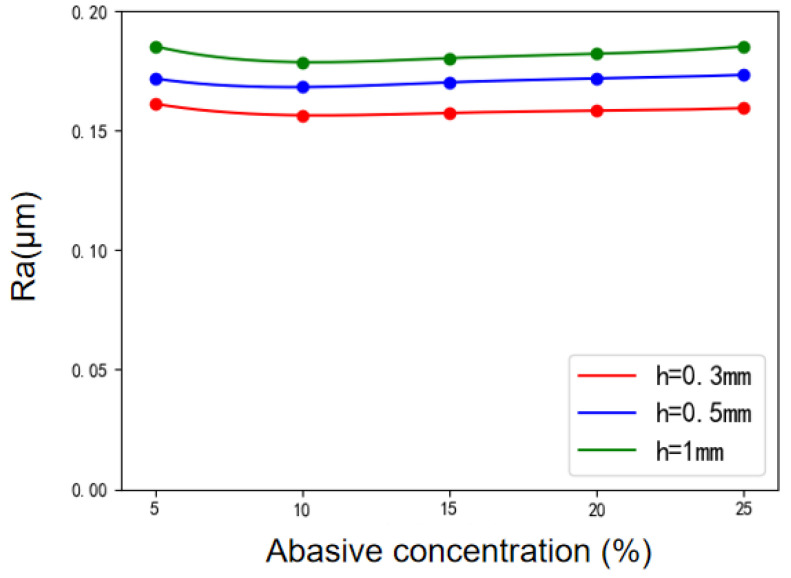
Variation of surface roughness with abrasive concentration under various polishing gaps.

The variation trend of surface roughness with abrasive concentration under various polishing gaps is shown in Figure 7. When the abrasive concentration is about 10%, the surface roughness is the lowest, and the polishing effect is the best. However, the effect of abrasive concentration on surface roughness is not obvious generally. For a similar reason that the abrasive particle size has little effect on surface roughness, the polishing tool head used in the experiment is small, and the number of abrasive particles gathered at the end of the polishing tool head is also limited. Therefore, the improvement effect of abrasive concentration on surface roughness is not obvious.

**Figure 8:**
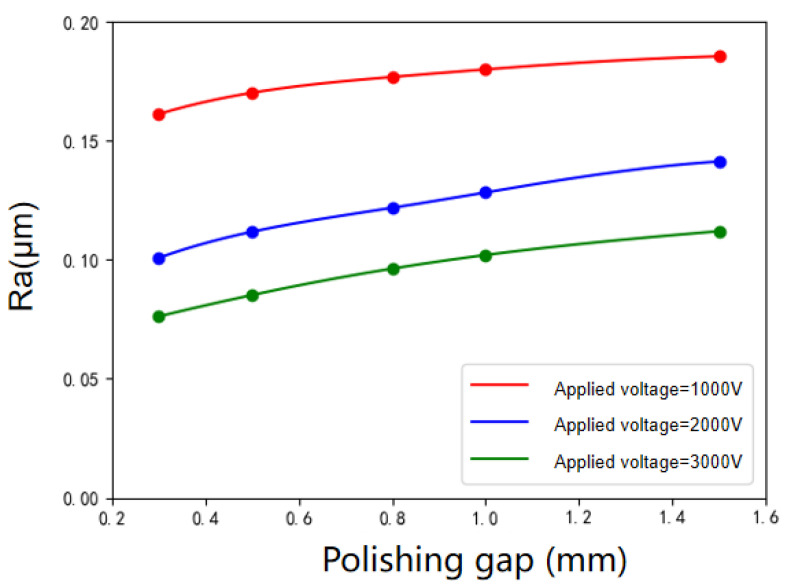
Variation of surface roughness with polishing gap under various applied voltages.

The variation of surface roughness of workpiece with polishing gaps under various applied voltages is shown in Figure 8. The polishing gap affects the electric field intensity in the polishing area, which directly affects the polishing effect of the workpiece surface. It can be seen that the surface roughness of the workpiece increases with the increase of the polishing gap. As the polishing gap increases fourfold from 0.3 to 1.5 mm, the surface roughness increases by 15.3% when the voltage is 1000 V and 42.5% when the voltage is 3000 V. The greater the distance from the end of the tool to the surface, the smaller the electric field intensity. The “hardness” of the polishing head formed by the ER effect is directly related to the electric field intensity. With the increase of the polishing gap, the “hardness” of the polishing head formed by the ER effect decreases gradually, and the material removal ability gets weaker. In addition, the larger the polishing gap is, the longer the chain which formed by the dispersed phase particles under the effect of the electric field is, and the arrangement is not as compact as that of the small gap. The abrasive particles cannot be attached to the chain better. During the process of tool spindle rotation, the abrasive particles may be far away from the polishing area, thus the material removal cannot be completed efficiently, and the surface roughness of the workpiece cannot be reduced.

**Figure 9:**
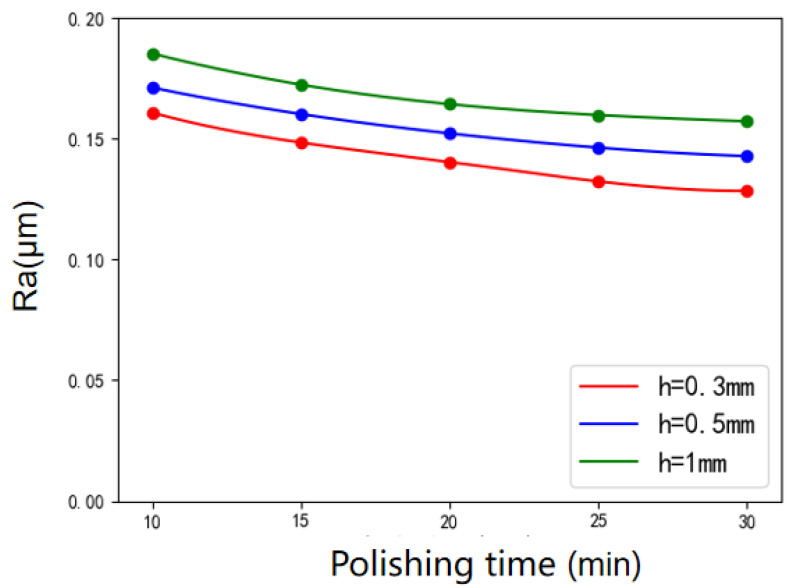
Variation of surface roughness with polishing time under various polishing gaps.

The variation of surface roughness of workpiece with different polishing times under various polishing gaps is shown in Figure 9. In general, the longer the polishing time, the lower the surface roughness and the better the polishing effect. The decreasing trend of surface roughness is most obvious at 20 min. With the increase of time, the surface roughness no longer decreases significantly, because it reaches the limit polishing effect that the polishing abrasive can complete. In order to reduce the surface roughness continuously and efficiently, we have to change other process parameters.

**Figure 10:**
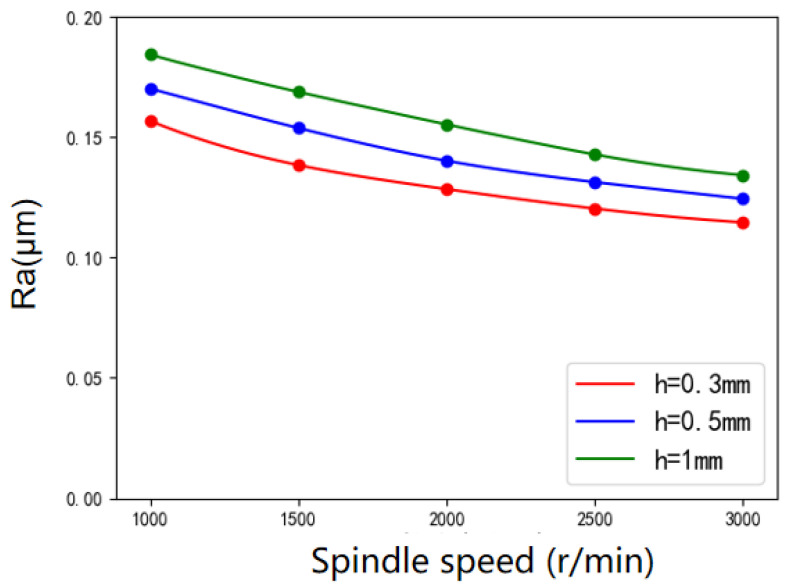
Variation of surface roughness with tool spindle speed under various polishing gaps.

Figure 10 shows the variation of workpiece surface roughness with tool spindle speeds under various polishing gaps. It can be seen that with the increasing of tool spindle speed, the surface roughness value of the workpiece decreases. When the spindle speed increases from 1000 to 3000 rpm, the surface roughness decreases by 34.4% when *h* = 0.3 mm and 26.5% when *h* = 0.5 mm. According to the hydrodynamics, the relative velocity between the polishing tool and the workpiece surface will increase with the increase of the polishing tool spindle speed. According to Preston law, the material removal rate will also increase. Therefore, increasing the spindle speed can effectively improve the polishing efficiency.

## 4. Conclusions

In this paper, a new ER polishing tool with an annular integrated electrode is developed. After determining the composition of ER fluid (the concentration of the dispersed phase and the type of the polishing abrasive), six possible factors affecting the ER polishing are determined: the applied voltage, the polishing abrasive particle size, the polishing abrasive concentration, the polishing gap, the polishing time and the tool spindle speed. Then, we get the best combination of parameters. In the optimized scheme selected, the applied voltage is set to 3000 V, the tool spindle speed is set to 3000 r/min, the polishing time lasts for 30 min, polishing gap is 0.3 mm, the abrasive particle size is selected as W10, and the abrasive concentration is 10%. According to the single factor experiments, the value of the surface roughness will decrease significantly with the increase of the applied voltage, the tool spindle speed and the polishing time and increase significantly with the increase of the polishing gap. The effect of the applied voltage, the tool spindle speed, the polishing time and gap on the surface roughness is obvious, but the effect of the polishing abrasive particle size and concentration on the surface roughness is relatively small.

## Figures and Tables

**Table 1:** Distribution of the factors and the levels in the experiments.

	A Applied Voltage	B Abrasive Particle Size	C Abrasive Concentration	D Polishing Gap	E Polishing Time	F Spindle Speed
	(V)	(μm)	(%)	(mm)	(min)	(r/min)
1	1000	1	5	0.3	10	1000
2	2000	5	10	0.5	20	2000
3	3000	10	15	1	30	3000

**Table 2:** Experimental scheme and results.

Experiment Number	Applied Voltage	Abrasive Particle Size	Abrasive Concentration	Polishing Gap	Polishing Time	Tool Spindle Speed	Roughness after Polishing
	(V)	(μm)	(%)	(mm)	(min)	(r/min)	(μm)
1	3000	5	5	0.3	30	3000	0.0325
2	3000	10	10	0.5	20	1000	0.0842
3	1000	5	15	0.5	10	1000	0.1702
4	3000	5	5	1	20	1000	0.1366
5	1000	10	15	1	20	3000	0.1268
6	2000	5	10	1	10	3000	0.0894
7	2000	5	15	0.3	20	2000	0.0823
8	3000	1	15	1	10	2000	0.0743
9	3000	1	15	0.5	30	3000	0.0452
10	3000	10	10	0.3	10	2000	0.0745
11	2000	10	5	0.5	10	3000	0.0882
12	1000	1	10	0.3	20	3000	0.1156
13	1000	5	10	0.5	30	2000	0.0908
14	2000	1	5	0.5	20	2000	0.0894
15	2000	1	10	1	30	1000	0.1263
16	2000	10	15	0.3	30	1000	0.1008
17	1000	1	5	0.3	10	1000	0.1612
18	1000	10	5	1	30	2000	0.1042
k1	0.128	0.102	0.102	0.094	0.11	0.13	
k2	0.096	0.1	0.097	0.095	0.106	0.086	
k3	0.075	0.096	0.1	0.11	0.083	0.083	
Range	0.053	0.006	0.005	0.016	0.027	0.047	

## Data Availability

Not applicable.
